# Use of static and dynamic [^18^F]-F-DOPA PET parameters for detecting patients with glioma recurrence or progression

**DOI:** 10.1186/s13550-020-00645-x

**Published:** 2020-05-29

**Authors:** Timothée Zaragori, Merwan Ginet, Pierre-Yves Marie, Véronique Roch, Rachel Grignon, Guillaume Gauchotte, Fabien Rech, Marie Blonski, Zohra Lamiral, Luc Taillandier, Laëtitia Imbert, Antoine Verger

**Affiliations:** 1 Department of Nuclear Medicine & Nancyclotep Imaging platform, Université de Lorraine, CHRU-Nancy, F-54000 Nancy, France; 2 IADI, INSERM, UMR 1254, Université de Lorraine, F-54000 Nancy, France; 3 INSERM, U1116, Université de Lorraine, F-54000 Nancy, France; 4 Department of Pathology, Université de Lorraine, CHRU-Nancy, F-54000 Nancy, France; 5 INSERM U1256, Université de Lorraine, F-54000 Nancy, France; 6 Department of Neurosurgery, Université de Lorraine, CHRU-Nancy, F-54000 Nancy, France; 7 Centre de Recherche en Automatique de Nancy CRAN, CNRS UMR 7039, Université de Lorraine, F-54000 Nancy, France; 8 Department of Neuro-oncology, Université de Lorraine, CHRU-Nancy, F-54000 Nancy, France

**Keywords:** [^18^F]-F-DOPA, Glioma, Recurrence, Dynamic analysis, Amino-acid PET

## Abstract

**Background:**

Static [^18^F]-F-DOPA PET images are currently used for identifying patients with glioma recurrence/progression after treatment, although the additional diagnostic value of dynamic parameters remains unknown in this setting. The aim of this study was to evaluate the performances of static and dynamic [^18^F]-F-DOPA PET parameters for detecting patients with glioma recurrence/progression as well as assess further relationships with patient outcome.

**Methods:**

Fifty-one consecutive patients who underwent an [^18^F]-F-DOPA PET for a suspected glioma recurrence/progression at post-resection MRI, were retrospectively included. Static parameters, including mean and maximum tumor-to-normal-brain (TBR) ratios, tumor-to-striatum (TSR) ratios, and metabolic tumor volume (MTV), as well as dynamic parameters with time-to-peak (TTP) values and curve slope, were tested for predicting the following: (1) glioma recurrence/progression at 6 months after the PET exam and (2) survival on longer follow-up.

**Results:**

All static parameters were significant predictors of glioma recurrence/progression (accuracy ≥ 94%) with all parameters also associated with mean progression-free survival (PFS) in the overall population (all *p* < 0.001, 29.7 vs. 0.4 months for TBR_max_, TSR_max_, and MTV). The curve slope was the sole dynamic PET predictor of glioma recurrence/progression (accuracy = 76.5%) and was also associated with mean PFS (*p* < 0.001, 18.0 vs. 0.4 months). However, no additional information was provided relative to static parameters in multivariate analysis.

**Conclusion:**

Although patients with glioma recurrence/progression can be detected by both static and dynamic [^18^F]-F-DOPA PET parameters, most of this diagnostic information can be achieved by conventional static parameters.

## Background

Gliomas represent approximately 80% of malignant tumors of the central nervous system (CNS) [1]. These tumors have a poor prognosis with a median overall survival of 15 months for glioblastomas, the most common of glioma entities. This particular poor prognosis partly results from a high risk of recurrence/progression with a median progression-free survival (PFS) of only 8 to 11 weeks for high-grade gliomas [2]. Even though magnetic resonance imaging (MRI) remains the gold standard for imaging these tumors, MRI is nonetheless dependent on the disruption of the blood–brain barrier and may hence be limited with regard to the differential diagnosis between residual tumors and post-therapeutic changes in tumors with suspected recurrence [3].

Amino-acid PET has been proposed as a criterion for detecting glioma recurrence by the Response Assessment in Neuro-Oncology (RANO) group [3] and has become a current indication for differentiating glioma recurrence from treatment-induced changes, as stated in the recent joint European Association of Nuclear Medicine (EANM)/European Association of Neuro-Oncology (EANO)/RANO practice guidelines [4].

[^18^F]-F-DOPA is an amino acid PET tracer for which the ability to diagnose glioma recurrence has been previously established [5–7]. In particular, [^18^F]-F-DOPA PET was found to detect glioma recurrence with an accuracy as high as 82% in a retrospective study of 110 patients, in which the lesion-to-normal-tissue ratio was additionally predictive of PFS [8]. More recently, in a study in which glioma recurrence was proven histologically, [^18^F]-F-DOPA PET accuracy was reported to reach 84%, using maximum [^18^F]-F-DOPA uptake as diagnostic parameter [9]. This accuracy for detecting glioma recurrence was even higher, reaching 96%, in a prospective, albeit smaller study of 28 patients [10].

However, all of the aforementioned [^18^F]-F-DOPA PET studies [5–10] were conducted while solely taking into account static [^18^F]-F-DOPA PET parameters, whereas information provided by dynamic parameters was not considered. It should be noted that such dynamic parameter-derived information has previously been studied in the assessment of glioma recurrence/progression with another widely used amino-acid radiotracer, O-(2-[^18^F]fluoroethyl)-L-tyrosine ([^18^F]-FET) [11–14], [^18^F]-FET, and [^18^F]-F-DOPA being known to present relatively equal performances [15]. Dynamic [^18^F]-F-DOPA parameters have also been used to distinguish high grade from low-grade recurrent gliomas with regard to the World Health Organization (WHO) 2007 classification [16]. Moreover, such parameters were recently found to allow characterizing the molecular features of gliomas according to the WHO 2016 classification [17]. However, it remains unknown whether dynamic parameters may also enhance the diagnostic accuracy of [^18^F]-F-DOPA PET prescribed for detecting glioma recurrence/progression.

The aim of the present study was thus to assess the performances of [^18^F]-F-DOPA PET with both static and dynamic parameters in detecting patients with progressive or recurrent glioma and for assessing further relationships with patient outcome.

## Materials and methods

### Patients

From October 2012 to October 2017, 245 patients referred to the Department of Nuclear Medicine at the CHRU Nancy for brain tumor assessment were investigated by [^18^F]-F-DOPA PET. Fifty-one of these patients were retrospectively selected on the basis that they had an initial history of surgically resected glioma and that the considered [^18^F]-F-DOPA PET had been prescribed for differentiating recurrence/tumor progression from post-therapeutic effects after a non-contributive MRI. As usual in our department, a minimum delay of 2 months is always respected between the surgery or end of radiotherapy and the performing of [^18^F]-F-DOPA PET in order to reduce the risk of [^18^F]-F-DOPA PET false positives [18].

In such patients, a clinical follow-up and MRI are systematically performed at least every 3 months or at shorter intervals as clinically indicated after surgery [8]. For the present study, the final diagnosis of glioma recurrence/progression at 6 months or for further assessment of survival was blinded from the [^18^F]-F-DOPA PET results and was based on current guidelines for which a recurrence/progression is the result of any new tumor or brain lesion at MRI and/or clear increase in tumor size or in contrast enhancement, and/or significant clinical deterioration, with all of these criteria not being attributable to other non-tumor causes and not due to steroid tapering [19–21]. For 4 patients having undergone stereotactic biopsy or open resection, the diagnosis of recurrence/progression was assessed histologically.

The assessment of [^18^F]-F-DOPA PET parameters for differentiating recurrence/progression from post-therapeutic effects was based on the evaluation of the previous criteria during a 6-month follow-up period. However, PFS and overall survival (OS) were calculated from the date of the PET exam to the date of definite diagnosis of progression and of death, respectively, with a minimum delay of 19 months of observation. The final date for reporting any event for PFS or OS was June 1, 2019.

The local ethics committee (Comité d’Ethique du CHRU de Nancy) approved the retrospective data evaluation on June 7, 2018, and authorization from the CNIL (National Commission on Information Technology and Civil Liberties) was delivered on June 25, 2018 (authorization n° R2018-11). This study complied with the principles of the Declaration of Helsinki. Informed consent was obtained from all individuals included in the study.

### Initial pathological grading of the gliomas

All cases were reviewed and classified according to the WHO 2016 classification from tumor samples provided by surgery or stereotactic biopsy [22]. IDH mutation status was assessed by immunohistochemistry with IDH1 R132H protein expression (Dianova, clone H09), or by Sanger sequencing in case of ATRX immunohistochemical loss without IDH1 R132H staining [23]. Tumors presenting oligodendroglial morphology or showing IDH mutation without ATRX loss were additionally tested for 1p/19qco-deletion using multiplex PCR fragment analysis (loss of heterozygosity), or comparative genomic hybridization [24].

### PET recordings and image reconstruction

[^18^F]-F-DOPA PET-computed tomography (CT) scans were obtained on a Biograph hybrid system involving a six-detector CT for attenuation correction (Biograph 6 True Point, SIEMENS, Erlangen, Germany). Patients were instructed to fast for at least 4 h with some patients also receiving Carbidopa administration 1 h prior to their exam (*n* = 17). The CT scan was recorded first and immediately followed by a 30-min 3D list-mode PET recording initiated during the bolus injection of 3 MBq of [^18^F]-F-DOPA per kilogram of body weight. The static PET images were reconstructed from the list mode data ranging from 10 to 30 min post-injection [4, 7], while the PET images reconstructed for dynamic parameters encompassed 6 consecutive frames of 20 s each followed by 28 frames of 1 min each. The choice of this acquisition time frame was based on previous studies performed with [^18^F]-FET PET (from 0 to 40 min post-injection with a reconstructed 20- to 40-min static image [25]) and on the maximum observed uptake of [^18^F]-F-DOPA in a PET study involving high-grade and low-grade gliomas (respectively 8 and 10 min post-injection) [26].

All static and dynamic images were reconstructed with an OSEM 2D algorithm (2 iterations, 21 subsets, 4-mm Gaussian post-reconstruction filter), corrected for attenuation, scatter, and radioactive decay, and displayed in a 256 × 256 matrix with 2.7 × 2.7× 3.0 mm^3^ voxels.

### Analyses of PET images

Regions of interest (ROIs) were placed on the static PET images using a dedicated software (DOSIsoft, Cachan, France). A spherical ROI of 2 cm diameter, centered on the maximum lesion uptake, was used for determining maximum and mean standardized uptake values (SUV_max_ and SUV_mean_, respectively). Tumor-to-striatum (TSR) and tumor-to-normal-brain (TBR) ratios were computed as SUV_mean_ or SUV_max_ of the lesion uptake divided by the SUV_mean_ of the striatum (TSR_mean_ and TSR_max_) or of normal brain (TBR_mean_ and TBR_max_). The SUV_mean_ from the striatum was obtained from the contralateral basal ganglia, which was segmented using a threshold of 65% of maximal uptake, while the normal reference brain SUV_mean_ was obtained with a crescent shape ROI (2× 8 cm) positioned on the semi-oval center of the unaffected contralateral hemisphere, including white and gray matter [4].

When no abnormal [^18^F]-F-DOPA uptake was detected, the ROIs of the potential residual tumor were placed at the site of maximal MRI abnormalities with a fused display of PET and fluid attenuation inversion recovery (FLAIR) MRI images [7].

As previously described [27], the metabolic tumor volume (MTV) was obtained through a 3D auto-contouring process with a threshold corresponding to the SUV_mean_ of the contralateral striatum.

In addition, time-activity curves, representing the evolution of the TBR_mean_ as a function of time (TAC_ratio_), were extracted with the PLANET® Dose software (DOSIsoft, Cachan, France) and with the ROIs previously placed on static images (see above). Each dynamic frame was previously registered on the CT images in order to take into account potential patient movements during acquisitions [28]. Two dynamic parameters were determined from fitted curves to overcome noise effects, using a method already validated for [^18^F]-FET in the same setting [29], namely, (i) time-to-peak (TTP), corresponding to the delay between the onset of the dynamic acquisition (time of tracer injection) and the time-point of the maximal TBR_mean_ value and (ii) slope, calculated with a linear regression applied from the 10th to 30th minute.

### Statistical analysis

Categorical variables are expressed as percentages and continuous variables as median and interquartile range due to the non-normality of variable distributions.

#### Recurrence/progression at 6 months follow-up

Univariate analysis was performed using Mann-Whitney tests applied between patients with glioma recurrence/progression and the remaining patients. In order to calculate diagnostic performances, the optimal threshold for each static and dynamic PET parameter was determined from ROC curves using the maximal value of the product of sensitivity and specificity. Thereafter, a multivariable logistic regression model with forward selection was performed for predicting patients with glioma recurrence/progression.

#### Progression-free survival (PFS) and overall survival (OS)

The dichotomized parameters, which were determined according to previously mentioned optimal thresholds, were used in survival analyses. For this purpose, univariate survival probabilities according to the Kaplan-Meier method with the log-rank test used and the hazard ratio interval of each parameter with its 95% confidence interval were calculated to compare survival curves.

*p* values obtained in univariate analysis as well as in survival analysis were adjusted using Benjamini-Hochberg correction in order to reduce the risk of false discovery [30]. *p* values lower than 0.05 were considered as significant.

Analyses were performed with SPSS (SPSS Statistics for Windows, Version 20.0. Armonk, NY: IBM Corp) and R (R Foundation for Statistical Computing, Vienna, Austria).

## Results

### Patient characteristics and follow-up data

The study population included 51 patients with a median age of 50.8 [44.4–59.0] years, 23 of whom were women. According to the 2016 WHO classification for gliomas [22], 8 gliomas (16%) had been initially classified as IDH-mutant astrocytomas (including 2 with anaplastic component), 6 (12%) as IDH-wildtype astrocytomas (including 2 with anaplastic component), 12 (24%) as IDH-mutant and 1p/19q co-deleted oligodendrogliomas (including 4 with anaplastic component), 22 (43%) as IDH-wildtype glioblastomas (GBM), and 3 (6%) as IDH-mutant GBM. Median delay times from the date of surgery to the [^18^F]-F-DOPA PET exam and from the date of the non-contributive MRI to the PET exam were 12.7 [5.9–23.5] months and 16 [7–30] days, respectively.

After the subsequent 6-month follow-up period, 34 patients were ultimately considered as having a recurrent or progressive glioma (3 IDH-mutant astrocytomas, 6 IDH-wildtype astrocytomas, 6 IDH-mutant and 1p/19q co-deleted oligodendrogliomas, 17 IDH-wildtype GBM and 2 IDH-mutant GBM) with 4 cases exhibiting a second recurrence and 2 cases a third recurrence. The remaining 17 patients were thus considered to exhibit only treatment-related changes at MRI. Patient characteristics are detailed in Table 1 as well as in the supplemental Table.

**Table 1:** Patient characteristics

*n* = 51	Value
Age (years)	
Median [range]	50.8 [21.2; 75.3]
Female gender *n* (%)	23 (45)
Primary histopathological type *n* (%)	
IDH-mutant astrocytoma	8 (16)
IDH-wildtype astrocytoma	6 (12)
IDH-mutant and 1p/19q co-deleted oligodendroglioma	12 (24)
IDH-wildtype glioblastoma	22 (43)
IDH-mutant glioblastoma	3 (6)
Histopathological WHO grade of the primary tumor *n* (%)	
II	18 (35)
III	8 (16)
IV	25 (49)
Primary treatment *n* (%)	
Surgery	15 (29)
Surgery + radiotherapy	2 (4)
Surgery + chemotherapy	9 (18)
Surgery + radiotherapy + chemotherapy	25 (49)

*WHO* World Health Organization

With regard to the location of tumor recurrence, 94% (32/34) were observed in the area of the resection cavity, whereas the remaining 6% (2/34) were located remotely.

During an observation period of 41 [23–50] months following the PET exam, 22 patients (43%) died and 43 (84%) had evidence of tumor progression. In the overall population, median survival was 24 [14–43] months.

### PET prediction of glioma recurrence/progression

As detailed in Tables 2 and 3, all studied PET parameters, except TTP, were significant univariate predictors of glioma recurrence/progression (all adjusted *p* < 0.001), with a global diagnostic accuracy of 96% being reached with TBR_max_, TSR_max_, and MTV. Meanwhile, the curve slope was the sole significant dynamic PET predictor, although its predictive value was somewhat lower than that obtained with the other PET predictors (i.e., with a lower area under the ROC curve and with a global diagnostic accuracy of only 76.5%, as detailed in Table 3).

**Table 2:** Median [interquartile range] of PET parameters in the overall population as well as comparatively between the 2 groups of patients with or without glioma recurrence or progression

Parameter	Overall	No recurrence/progression (*n* = 17)	Recurrence/progression (*n* = 34)	Adjusted *p* value
Static				
TBR_max_	2.22 [1.36; 3.12]	1.26 [1.12; 1.37]	2.71 [2.20; 3.69]	< 0.001
TBR_mean_	1.57 [1.02; 2.13]	0.84 [0.75; 1.04]	1.95 [1.57; 2.70]	< 0.001
TSR_max_	1.31 [0.82; 1.90]	0.77 [0.65; 0.82]	1.64 [1.31; 2.28]	< 0.001
TSR_mean_	0.92 [0.65; 1.43]	0.51 [0.46; 0.65]	1.19 [0.92; 1.69]	< 0.001
MTV	1.49 [0.00; 8.22]	0.00 [0.00; 0.00]	5.35 [1.49; 15.75]	< 0.001
Dynamic				
TTP	7.70 [3.35; 18.65]	14.53 [1.55; 30.00]	7.67 [4.13; 14.62]	1
Slope	− 0.14 [− 0.82; 0.13]	0.07 [− 0.07; 0.31]	− 0.59 [− 0.94; − 0.08]	< 0.001

*MTV* metabolic tumor volume, *TBR* tumor-to-normal brain ratio, TSR tumor-to-striatum ratio

**Table 3:** Results provided by ROC curve analyses for the PET identification of patients with glioma recurrence or progression

	AUC	CI (95%) AUC	Threshold	Sensitivity	Specificity	Accuracy
TBR_max_	0.969	(0.923–1.0)	1.61	97.1%	94.1%	96.0%
TBR_mean_	0.983	(0.956–1.0)	1.3	94.1%	94.1%	94.1%
TSR_max_	0.976	(0.939–1.0)	1.0	97.1%	94.1%	96.0%
TSR_mean_	0.986	(0.964–1.0)	0.83	91.2%	100%	94.1%
MTV	0.978	(0.939–1.0)	0.045 mL	97.1%	94.1%	96.0%
Slope	0.818	(0.702–0.935)	− 0.26 h^−1^	67.6%	94.1%	76.5%

*AUC* area under the curve, *CI* confidence interval, *MTV* metabolic tumor volume, *TBR* tumor-to-normal brain ratio, *TSR* tumor-to-striatum ratio

On multivariate analysis, TSR_max_ was the only parameter selected by the model to predict glioma recurrence/progression, with no other dynamic or static PET parameter able to provide any significant additional predictive information (Table 4).

**Table 4:** Results of logistic regression for the prediction of recurrence/progression at 6 months after [^18^F]-F-DOPA PET

Parameter	Coefficient	*p* value
Intercept	− 10.179	0.001*
TBR_mean_	–	0.244
TBR_max_	–	0.605
TSR_mean_	–	0.116
TSR_max_	10.039	0.002*
MTV	–	0.729
Slope	–	0.380

*Indicates parameters ultimately included in the final multivariable model

*MTV* metabolic tumor volume, *TBR* tumor-to-normal brain ratio, *TSR* tumor-to-striatum ratio

**Fig. 1:**
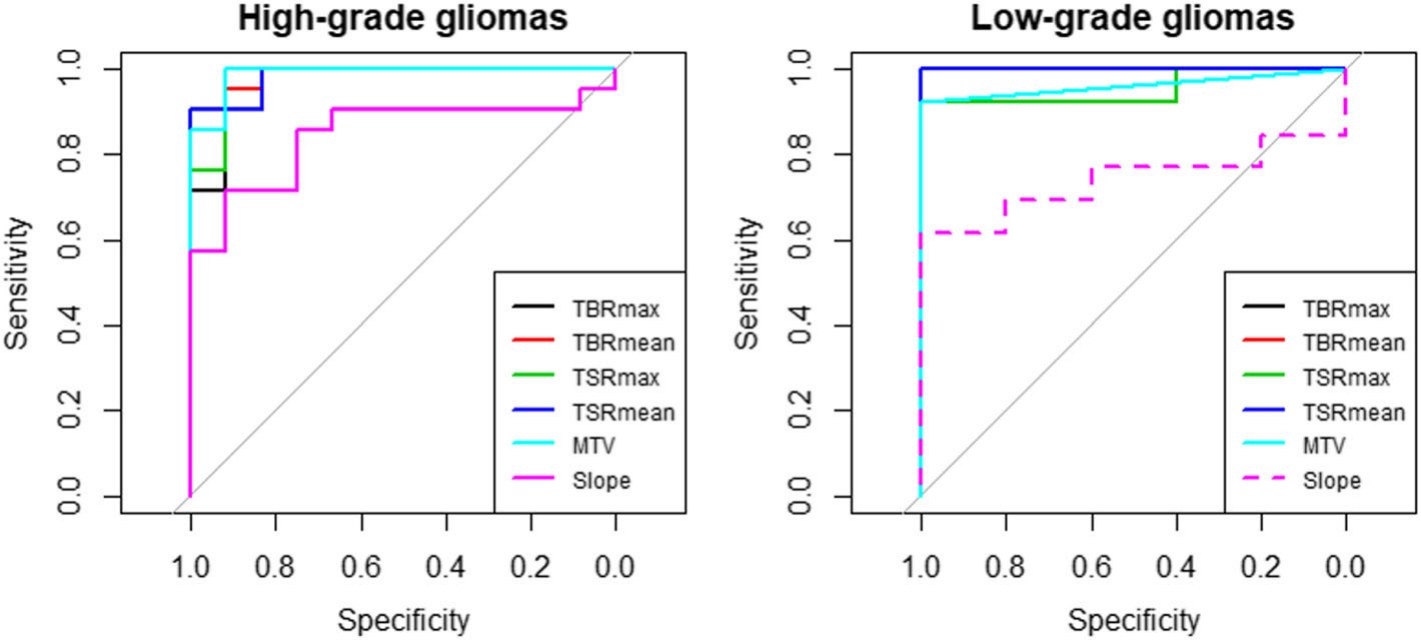
Receiver operating characteristic (ROC) curves for TBR_max_, TBR_mean_, TSR_max_, TSR_mean_, MTV, and slope parameters for differentiating between recurrent or progressive gliomas and treatment-related changes in high-grade gliomas (*n* = 33, left panel) and in low-grade gliomas (*n* = 18, right panel). Significant ROC curves are represented in solid lines

When separating the gliomas into two groups according to their respective grade at initial diagnosis [22], all of the static parameters as well as the curve slope were able to discriminate recurrent or progressive gliomas in initially classified high-grade gliomas (*n* = 33, *p* ≤ 0.001), whereas the slope parameter was not discriminative in low-grade gliomas (*n* = 18, *p* = 0.13) (Fig. 1).

### PET parameters for predicting PFS and OS

**Fig. 2:**
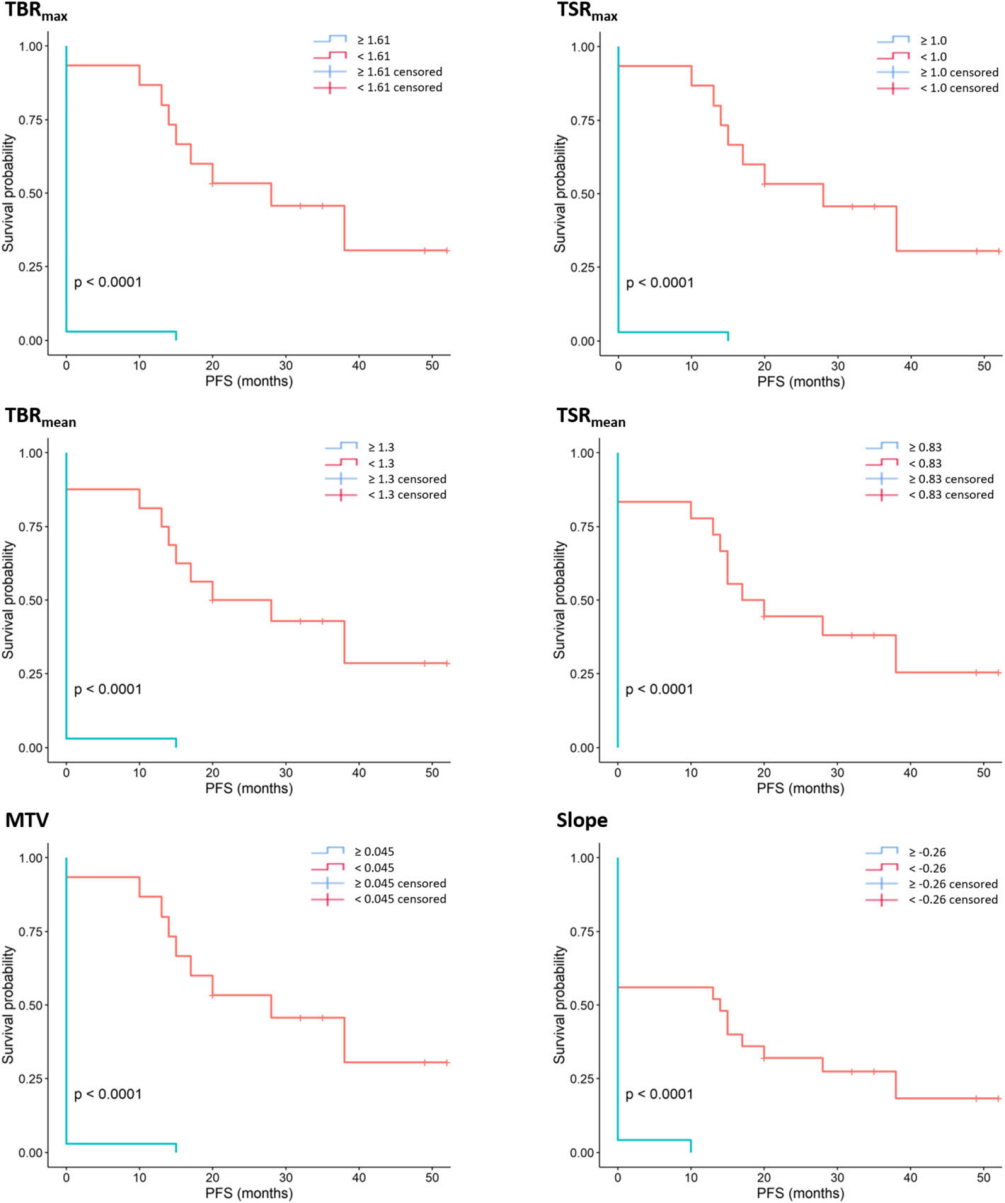
Kaplan-Meier survival plots for the prediction of progression-free survival using maximal tumor-to-background ratios (upper left panel), mean tumor-to-background ratios (middle left panel), metabolic tumor volume (lower left panel), maximal tumor-to-striatum ratio (upper right panel), mean tumor-to-striatum ratio (middle right panel), and slope (lower right panel) as discriminators. Corresponding log-rank test adjusted *p* values are < 0.0001 for all presented curves

All PET parameters, except TTP, were also significant predictors of PFS on Kaplan-Meier analyses, although none were predictive of OS (adjusted *p* value = 1). More precisely, PFS times were much longer in patients with vs. those without a TBR_max_ ≤ 1.61 (29.7 vs. 0.4 months, log-rank test adjusted *p* < 0.001, hazard ratio (HR) = 7.45 [2.39; 23.21], *p* < 0.01), a TBR_mean_ ≤ 1.3 (27.8 vs. 0.5 months, log-rank test adjusted *p* < 0.001, HR = 5.81 [2.04; 16.53], *p* < 0.01), a TSR_max_ ≤ 1.0 (29.7 vs. 0.4 months, log-rank test adjusted *p* < 0.001, HR = 7.45 [2.39; 23.21], *p* < 0.01), a TSR_mean_ ≤ 0.83 (25.6 vs. 0.0 months, log-rank test adjusted *p* < 0.001, HR = 6.0 [1.83; 19.63], *p* = 0.01), a MTV ≤ 0.045 mL (29.7 vs. 0.4 months, log-rank test adjusted *p* < 0.001, HR = 7.45 [2.39; 23.21], *p* < 0.01), or a curve slope ≥ − 0.26 h^−1^ (18.0 vs. 0.4 months, log-rank test adjusted *p* < 0.001, HR = 2.45 [1.18; 5.07], *p* = 0.03). The corresponding survival curves are depicted in Fig. 2.

**Fig. 3:**
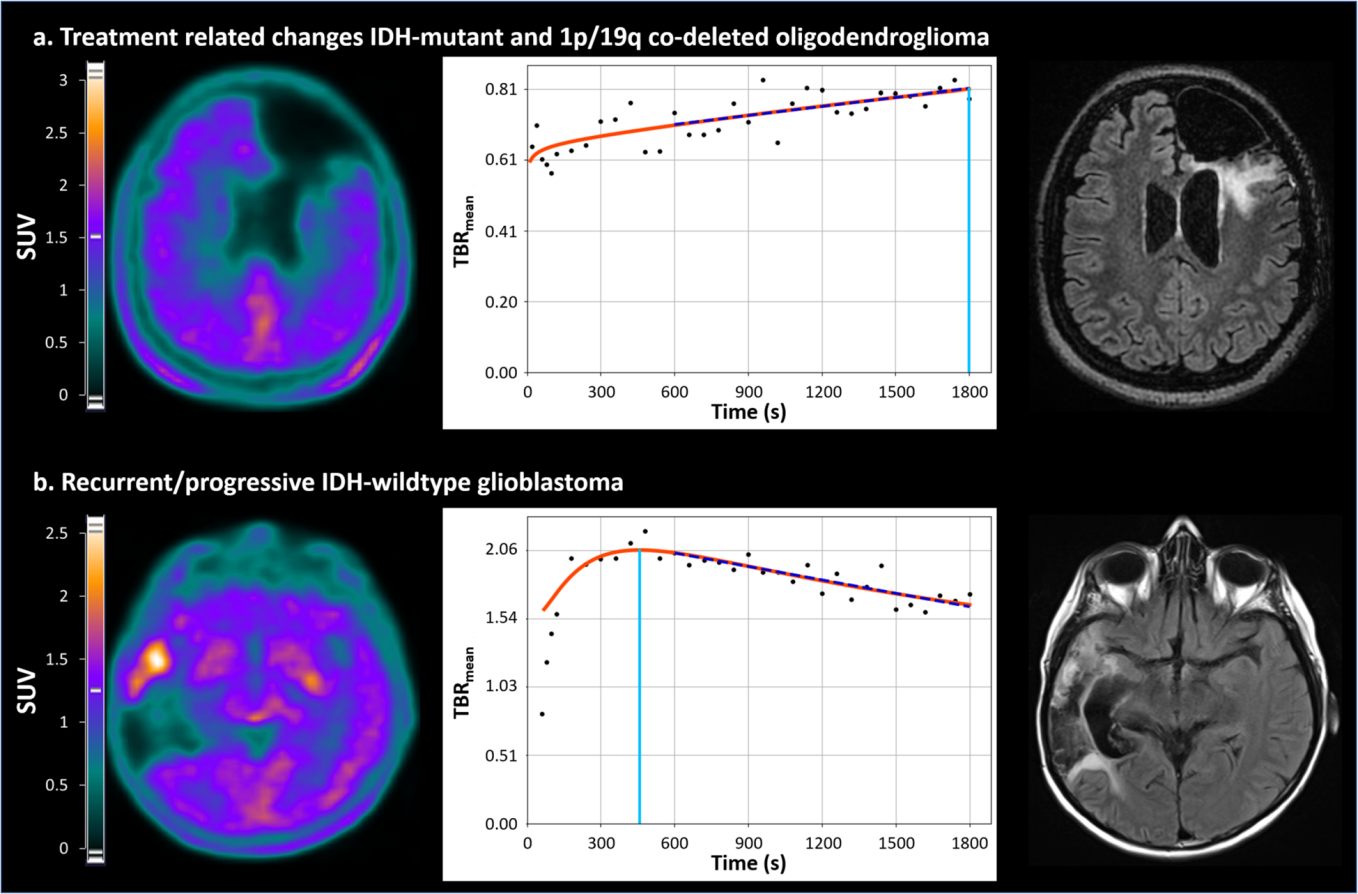
Representative examples of patients with or without glioma recurrence/progression investigated with [^18^F]-F-DOPA PET imaging, with axial slices of [^18^F]-F-DOPA PET (left column), dynamic TBR_mean_ curves (middle column) providing the time-to-peak delay-time (light blue dotted line), and the 10-to-30 min slope (dark blue dotted line), along with, for illustrative purposes, the same slice location recorded on a FLAIR MRI sequence (right column). **a** 51-year-old woman with no recurrent or progressive glioma (TBR_mean_ = 0.8, TBR_max_ = 1.1, TSR_mean_ = 0.5, TSR_max_ = 0.7, MTV = 0 mL, TTP = 30 min, and slope = 0.31 h^−1^). **b** 46-year-old woman with a progressive IDH-wildtype glioblastoma (TBR_mean_ = 1.9, TBR_max_ = 2.5, TSR_mean_ = 1.2, TSR_max_ = 1.6, MTV = 6.13 mL, TTP = 7.6 min, and slope = − 1.22 h^−1^)

Representative examples of patients with or without glioma recurrence/progression investigated with [^18^F]-F-DOPA PET imaging are provided in Fig. 3.

## Discussion

In the present population of patients with suspected glioma recurrence/progression at post-resection MRI, both static and dynamic [^18^F]-F-DOPA PET parameters were significant predictors of a glioma recurrence/progression at 6 months, as well as of progression-free survival on the longer term. This diagnostic information was however mostly achieved with conventional static parameters, with limited additional diagnostic information provided by dynamic parameters, contrary to that previously reported in the very different clinical setting of newly diagnosed gliomas [17].

The diagnostic performances of [^18^F]-F-DOPA PET imaging in the present study were very high, in keeping with those reported in a previous study [10], reaching a global accuracy of 96% for predicting patients with glioma recurrence/progression at 6 months after the [^18^F]-F-DOPA PET exam. This global accuracy is moreover higher when compared with the 82% level previously reported for the [^18^F]-F-DOPA PET detection of glioblastoma recurrence in a population of 110 patients [8].

Of particular note, upon multivariate analysis, none of the dynamic parameters were shown to provide any additional diagnostic information, comparatively to that obtained with static parameters. This statement may be explained by the high performance values obtained with the static parameters in this setting of suspicion of glioma recurrence/progression (accuracy ≥ 94%).

These results partially support previous results documented for [^18^F]-FET and in which the univariate predictive values of the dynamic parameters were consistently lower than that of static parameters [11–14]. In particular, in a large study cohort including 124 patients, dynamic [^18^F]-FET PET parameters (TTP, curve pattern) were less predictive than static parameters (TBR_max_, TBR_mean_) for glioma recurrence/progression [11].

Furthermore, in our study, the curve slope was predictive of outcome only in the subgroup of high-grade gliomas, similarly to what has been reported for dynamic parameters in a [^18^F]-FET PET study involving only high-grade gliomas [31]. Despite the fact that the number of patients was relatively limited, particularly for low-grade gliomas, this finding further strengthens the fact that dynamic parameters are ostensibly more relevant in instances of high expected uptake, i.e., in high-grade gliomas [5–7], in which a clear decreasing slope in kinetics is typically reported in these most aggressive glioma entities [16, 17]. The washout observed in high-grade gliomas, even if still to be well defined, could be related to microvessel density and LAT1 expression but also to the disruption of the blood brain barrier which likely facilitates the initial tumor uptake of the tracers, as well as their subsequent passive backdiffusion [32]. By contrast, lower-grade gliomas preferentially exhibit consistently increasing curves [17, 33, 34], which are very similar to the [^18^F]-F-DOPA time-activity curves expected in normal brain tissue or reactive tissue changes [5]. Thus, the use of dynamic parameters in this setting of low-grade gliomas could hence be more challenging. Another potential rationale is that a sufficiently high tumor uptake is required for an accurate determination of dynamic parameters, whereas this uptake was too low in the present study in cases of an absence of any glioma recurrence/progression (median TBR_mean_ = 0.84, see Table 2). In contrast to the previous hypotheses, the potential confounding influence of reactive tissue changes, in line with the inflammatory and healing processes induced by surgery and/or radiotherapy and/or chemotherapy [35, 36] cannot be used to explain the lower diagnostic performances from dynamic parameters. Both static and dynamic parameters should normally be affected in the same manner by the complex histology of the treated sites, involving varying levels of reactive gliosis, inflammatory cells, and radiation-induced changes. Moreover, in our clinical practice, [^18^F]-F-DOPA PET for detecting glioma recurrence/progression is performed at least 2 months after the surgery or at the end of radiotherapy, limiting the risk of the aforementioned confounding factors.

[^18^F]-F-DOPA PET imaging was also predictive of PFS in the present series of suspected recurrent gliomas, with a lower uptake and an increasing slope being associated with a longer PFS (Fig. 1). Several studies have observed similar relationships between static amino-acid PET parameters and PFS in recurrent gliomas [8, 37–40]. Irrespectively, the data provided herein by [^18^F]-F-DOPA PET imaging was furthermore predictive of patient outcome well beyond the 6-month period, reaching up to 18 and 29 months of mean PFS according to dynamic and static parameters, respectively.

The main limitations inherent to this study are that results were obtained retrospectively in a single center. In addition, our sample size was too limited for providing separate analyses according to the different molecular features of the gliomas involved in the present study, whereas it is possible that [^18^F]-F-DOPA uptake may vary according to these features [41–44]. Furthermore, the relative low number of histologically verified cases should lead to consider with caution the very high accuracy reported in this study. The time activity curves of tumors were expressed through ratios with tracer activity from normal brain, a method successfully tested by our team for the detection of newly diagnosed gliomas [17], along with the necessity to lower the possible interference of Carbidopa premedication [45]. Lastly, the calculation of the PFS and OS may be questioned given that all included gliomas, mixing several entities and especially low- and high-grade gliomas, may have benefited from different therapeutic strategies. Notwithstanding, all applied treatments were performed in keeping with general standards [20, 21].

## Conclusion

In summary, this novel study, assessing the relevance of [^18^F]-F-DOPA kinetics in the diagnosis of recurrent gliomas shows that patients with a glioma recurrence/progression, occurring remotely after surgery, may be detected by both static and dynamic [^18^F]-F-DOPA PET parameters. However, in this population mixing both low- and high-grade gliomas, much of this diagnostic information is achievable by conventional static parameters, contrary to that previously documented for the [^18^F]-F-DOPA PET detection of newly diagnosed gliomas. Further studies are warranted to investigate the relevance of such [^18^F]-F-DOPA kinetics in populations involving only high-grade gliomas.

## Supplementary information

**Additional file 1.** Supplemental Table.

## Data Availability

All data generated or analyzed during this study are included in this published article and its supplementary information files.

## Abbreviations

PFS: Progression-free survival
RANO: Response Assessment in Neuro-Oncology
EANM: European Association of Nuclear Medicine
EANO: European Association of Neuro-Oncology
WHO: World Health Organization
OS: Overall survival
CT: Computed tomography
ROI: Region of interest
SUV: Standardized uptake values
TBR: Tumor-to-normal-brain ratio
TSR: Tumor-to-striatum ratio
FLAIR: Fluid attenuation inversion recovery
MTV: Metabolic tumor volume
TTP: Time-to-peak
GBM: Glioblastomas
MET: ^11^C-methyl-methionine
